# Using artificial intelligence to make feeding and management decisions in dairy herds

**DOI:** 10.1093/af/vfaf059

**Published:** 2026-01-08

**Authors:** Alex Bach

**Affiliations:** Department of Animal Science, University of Lleida, Lleida, 25198, Spain; Agrotecnio, Lleida, 25198, Spain

**Keywords:** algorithm, data science, machine learning, optimization

Implications

- Developments in data science and improved computing power represent an opportunity for the dairy sector to use artificial intelligence (AI) to forecast animal responses.
- The combination of data from all aspects of the farm (nutrition, economics, genetics, reproduction, health, production, environment, and management) with novel computing algorithms may help to improve the nutritional status of cows and the profits of dairy herds.
- These novel models offer increased precision in immediate animal responses compared with factorial or mechanistic models, but lack the ability to explain or describe the cause of the predicted response.
- With adequate data, machine learning algorithms can assist nutritionist to define the optimum amount and combination of ingredients (and their nutrients) to maximize an outcome (i.e., profits, milk yield, reproductive performance…), or assist dairy producers to decide when to move cows between different production groups (and rations).

## Introduction

Milk production per cow has steadily improved through the implementation of new knowledge from research on nutrition, physiology, health, management, and genetics. Despite this progress, the dairy production sector remains subject to considerable economic and environmental challenges due to relatively high volatility in the feed ingredient and milk markets and extreme weather events (i.e., heat waves). Furthermore, the continuous progress in milk production is challenging the ability of the cow to consume sufficient nutrients to sustain all physiological functions. These conditions emphasize the need to improve diet formulation and accuracy of the models used to predict both requirements and animal response. New developments in data science and, especially, computing power, represent an opportunity for the dairy sector to use artificial intelligence (AI) to forecast animal responses from different inputs ranging from nutrients and ingredients to management. The animal science arena has been progressively implementing and testing a wide range of relatively novel AI algorithms. Most of these efforts have been recently summarized by Tedeschi and Menendez (2025). This article focuses specifically on the potential practical applications of AI, and its limitations, to make feeding and managing decisions in dairy cattle.

### The first step: data integration

A substantial number of farms have undergone digitalization and now possess extensive technical datasets containing information about virtually every aspect of dairy production. The combination and integration of these data is the first limiting step to facilitate the implementation of novel computing algorithms to improve nutrition, management, and economics of dairy herds. However, these data are often stored across different platforms or software systems, and the consolidation and integration of this information into a single database or platform that would allow holistic and prioritized decision-making remains challenging. In practice, most of these data integration is performed manually, and often partially, using spreadsheets. This approach is costly and time-consuming (data are rarely updated daily) and prone to errors (due to manual data entry). Consequently, farm management often relies on retrospective assessments, comparing present indicators with those from the previous month or with the same month in the preceding year. Such retrospective comparisons are inherently risky and ineffective due to the high variability across time in factors influencing profitability on dairy farms. The limitations of data integration are progressively, but slowly, overcome, with some examples being already implemented in the field such as DairyBrain (Cabrera and Fadul-Pacheco, 2021) or algoMilk (www.algoMilk.com) since 2020. Nevertheless, the considerable resistance from different companies to make data from sensors (i.e., milk meters, electronic scales for body weight, scales for mixing wagon, motion and activity monitors, etc…) easily accessible to producers and end-users to build more comprehensive and holistic datasets represents one of the largest limitations to implement AI in dairy production. Data from sensors or other sources, in isolation, have little value to improve the herds, for example, accumulating milk performance values in a database will rarely help to improve yield, as production depends on variables that are not in the database (i.e., feed intake, stocking density, weather conditions…). Similarly, body weight data alone is of little use, but if combined with precision feeding and milk production it becomes a valuable information (Brennan et al., 2023). Also, recording health events and acting on specific alarms will only assist producers in diminishing the negative impact of disease, but rarely will assist in tackling and solving the root of the problem (which would be the ultimate goal) unless they are combined with nutritional and management inputs.

### Artificial intelligence

The term of AI was first proposed in a scientific congress where it was defined as “*the ability of a machine to perform cognitive functions that we associate with human minds, such as perceiving, reasoning, learning, interacting with the environment, problem solving, decision-making, and even demonstrating creativity*” (McCarthy et al., 1956). Today, AI and machine learning (ML) are commonly used indistinctly, which may lead to misunderstandings or untruthiness (Thiebes et al., 2021) and create false expectations. Machine learning is a type of AI, but there are AI models that do not rely on ML (Kühl et al., 2021). Machine learning algorithms build a mathematical model using large sets of data inputs and outputs to recognize patterns and effectively “learn” or predict an outcome. The definition of “large” or “big” is arbitrary, but in general, when considering feeding and milking data, it is difficult to compile enough observations from a single herd to be considered “big data”. In addition of size, or number of observations, the data must be granular (Cirillo et al., 2021) or show a relatively large degree of variation.

Machine learning algorithms are classified as unsupervised, supervised, and reinforced. Unsupervised learning involves methods that aim to reveal previously unknown patterns in a dataset (Wang et al., 2009), an example of such models includes principal components and clustering analyses. Supervised learning is the most common in animal science and aims at predicting an outcome by applying an algorithm to a set of observations (Hastie et al., 2017). Supervised learning relies on many iterations on the same data to maximize a goal (i.e., the accuracy in the prediction) using neural networks, random forest, or gradient boost, among others. After sufficient repetitions and modification of the weights in the algorithm, the machine predicts an output upon receiving an input. These models are then cross-validated using fragments of the dataset that were not initially used during the training process (Morota et al., 2018), but ideally, they should also be validated using external datasets (Menendez et al., 2022) to avoid overfitting. (i.e., modeling underlying noise), which can be avoided ensuring that models are validated with external data regularly (Lever et al., 2016). Lastly, reinforced learning is similar to supervised learning, but it adds rewards and penalties allowing the model to continuously learn over time to increase the cumulative reward based, for instance, on animal responses (i.e., milk yield). This type of learning, as discussed later herein, is useful to design concentrate supplementation schemes (i.e., precision feeding) in parlors, milking robots, or concentrate dispensers. Some ML models can be extremely powerful, such as those based on large language models that lay behind generative AI applications. These models, rely on thousands of millions of inputs, a number that will likely never be reached in animal science.

**Figure 1.**
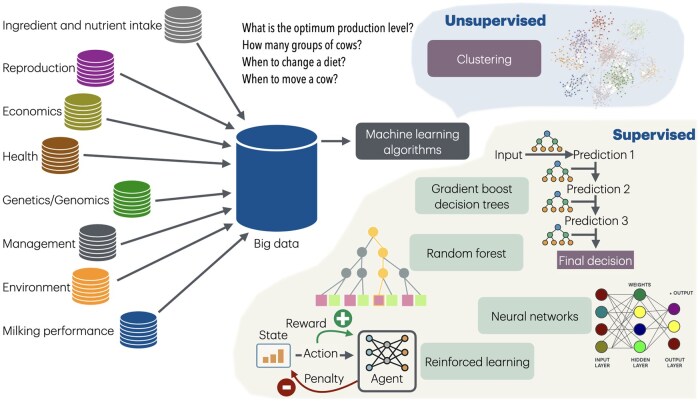
Schematic representation of data integration to generate a big database to run machine learning algorithms and address key questions in dairy production.

Geneticists pioneered the use of ML in animal science when using genomic data to predict phenotypes (Long et al., 2007). However, in the nutrition domain, combining data from different herds is usually necessary to build a sufficiently large database to effectively run ML algorithms. There are ML models that can be ensembled together, to build a unique prediction using multiple predictive functions. This approach is known as transfer or hierarchical learning. With hierarchical learning models, an algorithm is trained across different data sources (i.e., different herds) to build a large model describing general aspects of dairy production such as lactation curve, responses to different nutrients, responses to different stocking densities, etc.… and then a final prediction is made assembling a broader model with a more specific model derived from data of a particular herd. This approach is best suited to make predictions about dairy cow responses to different diets because it allows combining data from several herds relatively easily and overcomes some of the limitations associated with small datasets (i.e., from a single farm). Decision trees and random forests (Breiman, 200) are well suited to be ensembled, which have the additional advantage that they can easily accommodate nonlinearity (such as diminishing returns in nutrient absorption with increased feed intake). Neural networks and support vector machines are other types of ML that also well suited to build ensemble models, but bagging these ML is less frequent and a bit more complicated. Complex questions, such as determining the optimal production level at which to formulate diets to maximize economic returns, when to move cows from different nutritional groups, deciding how many nutritional groups are optimal in a herd, etc.… become more tractable through the integration of on-farm data streams and the application of ML algorithms (Figure 1).

### State-of-the-art of diet formulation

Formulating and feeding a well-balanced diet ensures optimal milk production and quality (Gross, 2022), health (Cardoso et al., 2020), reproductive performance (Bach, 2018), and ultimately may contribute to a more efficient use of natural resources. In addition, feeding is the most important contributor to the overall cost associated with dairy production, and thus, developing and implementing models that can accurately predict animal responses based on feed supply (and its corresponding investment) has been one the most significant drivers to improve production, health, and profits in dairy farms. Historically, diet formulation strategies have relied on defining a “reference cow” with an estimated, or often imaginary, body weight, days in milk, milk production and composition, and then forecast feed intake and nutrient requirements for a group of cows. Initially, these nutritional models were factorial in nature: they estimate nutrients produced or accumulated in the animal and then work backwards by applying factors related to digestibility and metabolic utilization to derive requirements to sustain specific physiological functions (i.e., maintenance, gestation, lactation) for a single cow. These models were constructed using equations based on associations (i.e., regression or linear equations linking inputs such as protein intake with outputs such as milk protein yield). Then models based on mechanistic functions (i.e., equations that attempt to describe responses based on expected physiological pathways) emerged, and nowadays most models used in the field such as NASEM (2021), the Cornell Net Carbohydrate and Protein System (Van Amburgh et al., 2015), or INRA (Noziere et al., 2018) use a combination of factorial and mechanistic approaches.

A limitation of the models currently used is that they include independent equations for each nutrient, and although some models attempt to account for nutrient interactions, in practice, each nutrient is governed by a distinct function. Consequently, when evaluating a diet to estimate milk yield, these models often predict different levels of milk yield depending on the nutrient that is considered. In addition, these models depend on a relatively large number of nutrients and nutritional characteristics from every ingredient composing the diet. Some of these nutrients (i.e., amino acids) and nutritional characteristics (i.e., digestion rates) are not commonly determined, and nutrient supply is usually derived from table values. Furthermore, the sampling process of ingredients along with the analytical procedures represent a large source of variation, and if ingredients are analyzed just once, analytical values may lead to an unneeded change of a diet or the formulation of a diet with an unbalanced nutrient supply (Weiss and St-Pierre, 2024). But, the lack of precision in estimating requirements and describing the nutrient values of each ingredient entails an economic loss, as nutritionists use safety margins (i.e., a greater than optimal provision of some nutrients) to compensate for the noise or variation in animal response and the knowledge gap about the real nutrient profile of all or some of the ingredients included in the diet.

Lastly, traditional models assume that all cows would respond, on average, in the same manner to a given nutrient supply, independently of their location, management, and other intangible factors (they target an “average” or “imaginary” cow). However, cows from different herds can respond differently, even when sharing similar genetic backgrounds, in terms of milk production when fed an exact same diet (Bach et al., 2008).

### Artificial intelligence to optimize diet formulation

Presently, it is possible to integrate diverse datasets with parameter observations that may influence milk production including genetics, nutrition, environment, health, and management, and employ ML to estimate nutrient requirements or, more effectively, to identify the optimal combination of on-farm ingredients to achieve maximal economic returns. For example, instead of formulating diets explicitly based on energy, amino acids, fiber, protein, starch, and minerals, ML can directly formulate diets using feedstuffs such as alfalfa, corn, or soybean meal, by integrating nutrient interactions within an “ingredient-based” framework. The process would be similar to the approach used in the INRA (Noziere et al., 2018) model to assign energy values to ingredients, where barley is given a value of 1, and then the other ingredients receive a greater o a lower value depending on the animal performance obtained when fed. Of course, the predictive ability of ML models improve as the number of parameters considered increases; thus, if the content of some measurable nutrients (i.e., DM, CP, NDF…) are provided for each ingredient, predictions improve.

**Figure 2.**
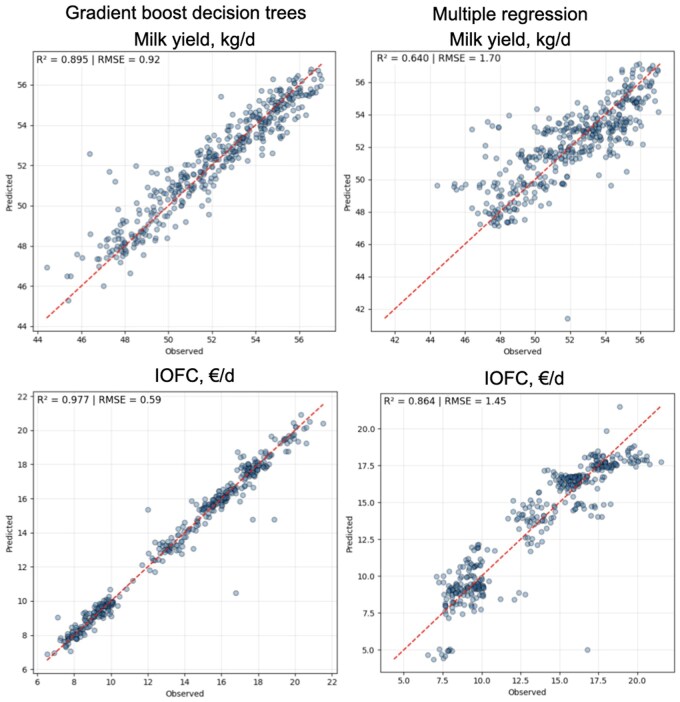
Observed vs predicted milk yield and income over feed cost (IOFC) obtained by machine learning (gradient boost decision trees) or multiple regression using an algoMilk dataset containing 2,073 daily averages of ingredient (and nutrient) intake, weather parameters, milk yield, milk composition, body weight, and IOFC from a pen of approximately 120 cows.

A pivotal advantage of being able to predict with increased precision immediate animal responses to a given diet is that nutrient supply can be targeted to maximize, not milk production, but economic returns, by considering nutrient profile, ingredient interactions, feed intake, animal responses, feed costs, and milk prices altogether. Furthermore, these predictions can be driven by data specific to a herd (or a group of cows) and can consider the intrinsic characteristics of a particular farm (or pen within a farm), which represents an advantage for models based on AI over traditional ones. Making a comparison with statistics, it would be like fitting a random effect for farm or pen when adjusting model responses to the peculiarities of that specific environment. This advantage allows tackling a key question that is often not adequately considered when formulating diets for cattle, which is: what level of milk production should a diet be formulated for. Traditionally, the dairy industry has operated under the principle that “*more milk equates to greater profitability*”, thus, nutritionist typically formulate diets for some additional liters above the average production of the herd (or group of cows). However, this assumption does not always hold true. There are two main reasons for this. On one side, feed efficiency follows the law of diminishing returns, and because marginal feed costs increase progressively with milk production, profits associated with improving milk yield may, in some cases, be considerably lower than expected (Bach et al., 2020). The second reason lies in the fact that cows are fed in groups. Within any given group, when additional investment in feed nutrients is made (i.e., by increasing dietary density), a subset of cows fails to respond with increased milk yield, especially if cows are beyond peak production. For non-responding cows, feeding a more expensive diet leads to economic losses as they consume more expensive feed without improving output. Only cows that respond with higher income over feed cost (IOFC) or gross margin once feed has been paid for, not only milk, generate additional profit. Algorithms based on ML can help in determining the optimum level of nutrients that should be supplied to a group of cows based on current milk price, feed costs, and an accurate estimation of animal responses (both in terms of yield and profits) to specific ingredients. As an example, data obtained from a farm using algoMilk containing 2,073 daily observations of ingredient (and nutrient) intakes, weather variables, milk yield, milk composition, body weight, and profits (IOFC) from a pen containing approximately 120 cows were used to predict yield and IOFC using a ML algorithm based on gradient-boost decision trees or a classic multiple regression analysis. Figure 2 shows the superiority of AI algorithms vs classical multiple regression illustrated as the relationship between observed and predicted milk yield and IOFC determined either using ML (*R*^2^ = 0.90 and 0.98; RMSE = 0.92 kg/d and 0.59 €/d, respectively) vs multiple regression (*R*^2^ = 0.64 and 0.86; RMSE = 1.70 kg/d and 1.45 €/d, respectively).

It is important to note that, even though a theoretical diet fed in a herd may not change over a period, the actual ingredient and nutrient composition changes daily due to errors during mixing (Bach, 2024). These errors or deviations are the base to provide granularity for the ML to be able to predict animal responses. By integrating changes in ingredient and nutrient supply with change sin animal performance along with other exogenous variables such as weather, stocking density, etc… a ML can be trained for example using gradient-boost decision trees. Once the model is trained, it can then be used to determine the exact combination of ingredients to maximize, not milk, but IOFC using for example sequential quadratic programming under equality (i.e., fixed dry matter intake) and inequality (i.e., ingredient and nutrient intake bounds) constraints to generate a ration that maximizes expect IOFC while preserving realistic dietary composition and intakes. Table 1 shows the resulting optimum diet that would maximize IOFC and compares it with the ration that was fed at the moment of running the model using the example dataset and described approach above. Similar results were reported by Nguyen et al. (2020) when comparing support vector machine, random forests, and neural networks, over conventional regression analysis using data from only 36 cows over a period of 10 months to predict individual milk yields depending on the amount of nutrients consumed.

**Table 1.** Comparison between actual and expected cow responses of a diet currently fed and a diet optimized using artificial intelligence

	Current	Optimized
Ingredients, kg as fed/d		
Corn silage	16.03	16.38
Sweet corn	9.79	9.82
Oats silage	6.56	6.57
Corn grain	5.90	5.82
Corn flakes	5.79	5.60
Soybean meal	3.21	3.1
Canola meal	2.60	2.87
Mineral-vitamin premix	1.30	1.28
Alfalfa hay	1.25	1.04
Wheat grain	1.0	1.0
Rumen inert fat	0.51	0.49
Rumen protected methionine	0.04	0.04
Nutrients		
Crude protein, %	16.3	16.4
Starch, %	31.2	30.9
Neutral detergent fiber, %	26.4	26.7
Forage, %	28.9	28.8
	Actual	Predicted
Dry matter intake, kg/d	28.5	28.3
Milk yield, kg/d	53.4	53.2
Income over feed cost, €/d	19.34	19.36
Difference in milk yield, kg/d	−0.166	
Difference in income over feed cost, €/d	+0.015	

The optimized diet was generated using a gradient boost decision tree model trained on 2,073 daily observations obtained from an integrated database (algoMilk) to predict animal response and finding the maximum economic return using sequential quadratic programming.

Machine learning algorithms are effective in making predictions, but traditional models (hybrids of factorial and mechanistic approaches) are much better suited to indicate the reasons driving changes in performance. However, this limitation of ML can be partially addressed by using techniques such as a Shapley additive explanations (Lundberg and Lee, 2017) or agnostic-permutations (Fisher et al., 2019), which allow to classify the different input variables by the relative impact on the output parameter.

### Artificial intelligence for precision feeding

Both nutrient requirements and feed intake of dairy cows change relatively frequent over time, which means that, ideally, diets (and nutrient supply) should be adjusted frequently. Even though traditional feeding systems (i.e., NASEM, 2021) count with submodels that consider the impact of environmental conditions on nutrient requirements, in practice these submodels are not fully implemented in the field, as diets are typically formulated less frequently than changes in environmental conditions. In addition to environmental changes, many other aspects, such as stocking density, silage quality, influence cow milking performance and should be considered when determining optimum nutrient supply. Machine learning algorithms provide enough flexibility to incorporate real-time variations in the input variables to make predictions at both individual and group levels, and can be effective, for example, to adjust nutrient inputs based on changes in environmental temperatures, or determining the amount of concentrate to be fed individually during milking in automatic milking systems, batch milking systems, conventional parlors, or concentrate dispensers.

Traditionally, concentrate supplementation is being performed based on parity, stage of lactation, and milk production, using a set of rules applied equally to all cows. But this approach has been shown to have little impact on milk production (Little et al., 2016), mainly because not all cows respond in the same manner to concentrate supplementation. Cows will reduce consumption of a partial mixed ration in the feed bunk between 1 and 1.6 kg for every kg of concentrate consumed while milking (Bach et al., 2007; Hare et al., 2018), and this seems to be affected by composition of both the partial mixed ration and the concentrate, but also on the stage of lactation, and individual animal differences.

Some authors have proposed alternative approaches based on historical performance using simple regression or even averages (Souza et al., 2025), directly based on individual nutritional needs estimated from individual intakes and milking performance (Morey et al., 2023), or based on more complex approaches such as random coefficient models (André et al., 2010). But implementing reinforced learning algorithms described above, and supplement individual cows based on their response rather than on a fixed feeding regime based on milk production and stage of lactation seems to be a perfect fit. Using reinforced learning cows that respond positively in milk yield (and more importantly economic returns) to concentrate supplementation can be rewarded with more concentrate, and those that do not can be penalized by maintaining or reducing their concentrate allowance.

### Artificial intelligence to define grouping strategies

**Figure 3.**
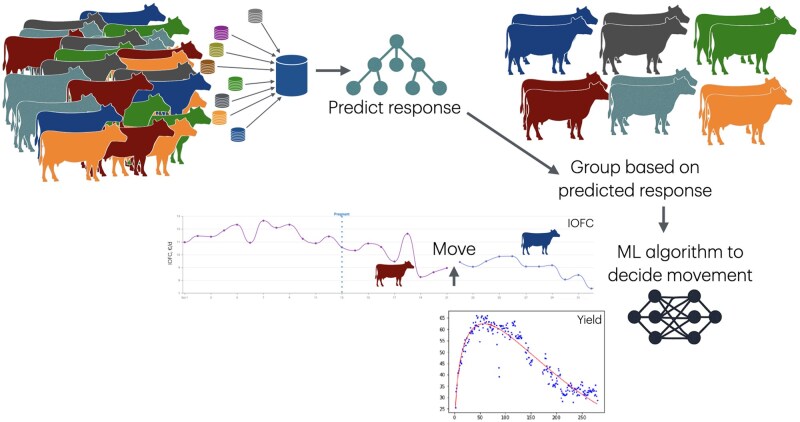
Schematic representation of the chain of events involved in using artificial intelligence to decide when to move cows from different feeding groups. First: data integration; second: predict animal response to different diets; third: group cows based on expected performance on the receiving diet, and fourth: decide when to make a movement.

Forming groups of cows according to their nutritional needs can lead to improved economic profits (St-Pierre and Thraen, 1999; Kalantari et al., 2016), but there is still a large number of herds that feed a single diet to all lactating cows, thus potentially losing an opportunity to not only ameliorating the environmental impact of dairy production, but also improving the health status of the cows and improving IOFC (Cabrera and Kalantari, 2016). The most common arguments for not implementing different groups (and diets) in dairy herds include concerns of losing milk production, difficulties of deciding when to move cows between different groups, and defining the target milk production to formulate the diets for each group. These concerns can be overcome by estimating changes in milk production when moving animals between pens and formulating the diets to ensure that the unit cost of nutrients is greater in the original than in the receiving diet (Bach, 2023). Making the decision of when to move cows is complex, and in practice, cows are often moved at suboptimal times (or simply not moved and fed a single diet for the entire lactation). Barrientos-Blanco et al. (2020) illustrated how using a combination of equations extracted from factorial models (i.e., NASEM, 2021) to estimate animal responses and intake along with an algorithm to rank cows according to their parity, stage of lactation and nutritional needs to form different groups of cows was more profitable than feeding a single diet for all cows. But this approach can be further refined by first predicting animal responses to different diets, using the approach described above involving gradient boost decision trees, and then grouping cows based on the expected response rather than relying on the performance on the current diet using unsupervised learning involving clustering methods. Once the ideal groups of cows are composed, then, in a second step, another ML algorithm can assist producers to detect the optimum moment to move a cow considering potential transient losses in milk yield and changes in IOFC by accounting for differences in nutrient supply and feed costs of the 2 diets involved in the movement. Figure 3 summarizes this process.

## Conclusions

The future of dairy production lies increasingly in the application of dynamic, farm-specific models over static or empirical models that assume all cows behave identically regardless of context (disregarding the effects of pen, farm, location, management….). The advantage of such approach over traditional one-size fits all models is that with sufficient data, the predictions for nutrient requirements and, especially animal responses, can be substantially improved and adapted to specific situations. However, these methods require the frequent data integration along with the development of relatively complex AI models.

*Conflict of interest statement*. Author declares no conflict of interest other than he was the founder of algoMilk.

## About the Author

**Alex Bach** is a research professor at the Department of Animal Science of the University of Lleida, Spain. Alex conducts research to understand the physiology and metabolism of cattle and developing models to improve nutrition and management of dairy herds to make them more sustainable. Dr Bach has more than 170 peer-reviewed publications covering aspects such as rumen and hindgut fermentation dynamics, protein degradation, amino acid metabolism, epigenetic implications of early life nutrition, long term effects of health issues in dairy replacements, cow responses to different management schemes, nutrition of calves and heifers, intake regulation and appetence, and concentrate provision and optimization with robotic feeding systems, and among others.
